# Analysis of a coupled fluid‐structure interaction model of the left atrium and mitral valve

**DOI:** 10.1002/cnm.3254

**Published:** 2019-12-23

**Authors:** Liuyang Feng, Hao Gao, Boyce Griffith, Steven Niederer, Xiaoyu Luo

**Affiliations:** ^1^ School of Mathematics and Statistics University of Glasgow Glasgow UK; ^2^ Departments of Mathematics, Applied Physical Sciences, and Biomedical Engineering University of North Carolina Chapel Hill North Carolina USA; ^3^ Department of Biomedical Engineering King's College London London UK

**Keywords:** atrial fibrillation, fibre‐reinforced hyperelastic material, fibre structure, fluid‐structure interaction, left atrial appendage, left atrium, mitral regurgitation, mitral valve

## Abstract

We present a coupled left atrium‐mitral valve model based on computed tomography scans with fibre‐reinforced hyperelastic materials. Fluid‐structure interaction is realised by using an immersed boundary‐finite element framework. Effects of pathological conditions, eg, mitral valve regurgitation and atrial fibrillation, and geometric and structural variations, namely, uniform vs non‐uniform atrial wall thickness and rule‐based vs atlas‐based fibre architectures, on the system are investigated. We show that in the case of atrial fibrillation, pulmonary venous flow reversal at late diastole disappears, and the filling waves at the left atrial appendage orifice during systole have reduced magnitude. In the case of mitral regurgitation, a higher atrial pressure and disturbed flows are seen, especially during systole, when a large regurgitant jet can be found with the suppressed pulmonary venous flow. We also show that both the rule‐based and atlas‐based fibre defining methods lead to similar flow fields and atrial wall deformations. However, the changes in wall thickness from non‐uniform to uniform tend to underestimate the atrial deformation. Using a uniform but thickened wall also lowers the overall strain level. The flow velocity within the left atrial appendage, which is important in terms of appendage thrombosis, increases with the thickness of the left atrial wall. Energy analysis shows that the kinetic and dissipation energies of the flow within the left atrium are altered differently by atrial fibrillation and mitral valve regurgitation, providing a useful indication of the atrial performance in pathological situations.

## Graphical abstract

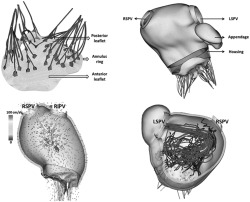

## 1 INTRODUCTION

The left atrium (LA) is a complex important chamber in the human heart. It consists of four components: septum, appendage, vestibule, and venous components.^1^ The septum is the shared wall between the left and right atrium, and left atrial appendage (LAA) is the tubular structure near the left pulmonary veins that connects to the LA main chamber and serves as a decompression chamber when the atrial pressure is elevated.^2^ The vestibule resembles the thin circumferential wall that connects the mitral valve (MV), a valvular structure that ensures the unidirectional flow from left atrium to left ventricle (LV). The majority of LA is the venous components that are connected to the pulmonary veins. Typically, there are four veins: the left superior and inferior pulmonary veins (LSPV/LIPV) and the right superior and inferior pulmonary veins (RSPV/RIPV). During the cardiac cycle, the LA receives oxygenated blood from the pulmonary veins during ventricular systole and acts as a reservoir; in early diastole, the LA serves as a conduit that allows blood to passively flow into the left ventricle; in late diastole, the LA contracts and pumps blood into the LV through the mitral valve. LA function can be described through stroke volume, conduit volume, and LA emptying volume, in which the conduit volume represents the blood flowing through LA during diastole and LA emptying volume is the LA cavity volume difference between end diastole and end systole.

LA mechanical dysfunction, for instance, in the presence of atrial fibrillation (AF), which causes an abnormal heart rhythm, can lead to reduced cardiac output,^3^, ^4^ thrombus formation,^5^, ^6^, ^7^ and higher stroke risk.^8^ Additionally, AF causes LA structural remodelling including atrial fibrosis,^9^ the thickening of LA wall,^10^, ^11^ and reduced atrial wall compliance.^12^ However, the effects of such wall thickness changes on the atrial haemodynamics have not yet been fully elucidated. On the other hand, LA function is also greatly affected by the MV through blood flow and fluid‐structure interaction (FSI). For example, in the case of mitral valve regurgitation (MVR), patients are found to have increased LA deformation and reduced contractile contribution^13^ and also atrial fibrillation.^14^

With the development of medical imaging techniques, methods such as transesophageal echocardiography (TEE), magnetic resonance imaging (MRI) 4D flow analysis, and tissue Doppler imaging (TDI) have been widely used to study the LA function under normal and pathological conditions.^15^, ^16^, ^17^ In addition, the LA function evaluation can also be achieved via multiphysics modelling which provides insights of the abnormal tissue behaviour such as atrial remodelling, the relation between large strain and development of fibrosis, and flow details that are important for improving surgical procedures.^18^, ^19^ Computational models with fluid structure interaction enable us to study both LA mechanical behaviour and haemodynamics under controlled conditions.^20^, ^21^, ^22^ More importantly, they provide tools for isolating the effects of different physiological and anatomical changes that occur under pathological conditions and for studying their impact on the link between LA and MV function.

To date, only limited progress has been made^23^, ^24^, ^25^, ^26^, ^27^, ^28^, ^29^, ^30^, ^31^ in multi‐physics modelling of the LA. This is a reflection of the complex anatomy and physiology of the LA. For example, LA has a complex myofibre structure composed of dominant muscle bundles that have different orientations across the LA wall,^1^, ^32^ such as the Bachmann's, Septopulmonary, and Septoatrial bundles. Therefore, it requires significant amount of work to define a physiologically detailed fibre structure for atrial material modelling. In addition, the LA is a thin‐walled structure with non‐uniform wall thickness.^10^ It is difficult to obtain accurate patient‐specific thickness measurements for LA geometry reconstruction. As a result, one simplification often made in LA modelling is the uniform wall thickness.^33^, ^34^

So far, few computational models have included detailed LA fibre models, and, consequently, the control of LA wall motion is often prescribed instead of driven by FSI. For instance, Menghini et al^23^ used an idealised LA geometry with prescribed wall motion and flow boundary conditions to study the fluid dynamics inside the LA. Koizumi et al^24^ applied controlled wall motion on patient specific LA geometries to study the haemodynamic changes in the case of AF. Besides the challenges mentioned in developing the LA‐only models, it is also important to include a physiologically realistic MV structure for a better understanding of the LA‐MV interaction and left heart function,^35^, ^36^, ^37^ especially in the case of MV dysfunction.^38^, ^39^, ^40^ However, due to the complex MV structure, researchers tend to use simplified outflow boundary condition to approximate the MV function. For instance, in the LA model developed by Koizumi et al,^24^ MV was assumed to open and close instantly, ignoring the leaflets motion. Similarly, Masci et al^26^ treated MV as an on/off switch in their fluid dynamics model of the LA. On the other hand, some effort has been made to explore the importance of the complex MV geometry in the case of mitral regurgitation, without involving a detailed LA model. For example, in the work by Einstein et al,^35^ a comprehensive strategy was proposed for a predictive analysis of mitral regurgitation with a Lagrangian segregated scheme for fluid‐structure interactions. Toma et al^41^ developed a patient‐specific mitral valve model with FSI and analysed the chordae rupture. They found that the MV coaptation line enclosed area depends on the ruptured chordae diameter, its location, and relationship with surrounding chordae. Caballero et al^42^ also investigated the effect of chordae rupture on left heart dynamics and found that the structure and strength of the regurgitant jet varies depending on the location and severity of the leaflets prolapse caused by the rupture.

The aim of the current study is to investigate how LA function varies under pathological conditions (eg, AF and MVR) and to study the effects of variations in geometric parameters, such as fibre structure and wall thickness, on the overall LA performance. To this purpose, we present a FSI model with an imaged‐based LA geometry and fibre structure, coupled with an MV model^43^ that includes physiologically realistic leaflets and chordae tendineae. The FSI analysis is implemented within an immersed boundary finite element framework (IB/FE),^44^ where the structures are modelled using hyperelastic constitutive laws. To our best knowledge, this is the first imaged‐based hyperelastic LA‐MV model with FSI that is used to study AF and MVR. A physiologically realistic LA model adapted from the previous work by Fastl et al^27^ is used in the current study. The LA model incorporates detailed fibre structures defined by an atlas‐based method,^27^ and it is then coupled to a MV model developed in the previous work.^43^ Investigations into LA function are then made under normal and pathological conditions using the coupled LA‐MV model. Furthermore, it is also used to study the effect of geometric variations (fibre structure and wall thickness) on the LA function.

The remainder of paper is organized as follows: Details of IB/FE method for FSI is described in Section 2. Structure model properties are discussed in Section 3. Implementation details and numerical results are presented in Sections 4 and 5. Finally, a discussion of the results is provided in Section 6.

## 2 METHODOLOGY

### 2.1 IB/FE formulation

The IB/FE formulation of FSI in this study follows the approach described in the work by Griffith and Luo,^44^ in which the deformation and elasticity of the structure are described in Lagrangian form, and the velocity, pressure, and incompressibility are described in Eulerian form. Let $\Omega \subset R^{3}$ denote the fixed physical domain occupied by the coupled fluid‐structure system and $\Omega_{0}^{s}\subset \Omega$ correspond to the initial Lagrangian domain for the structure. ***x***∈  are fixed Cartesian physical coordinates, $X\in \Omega_{0}^{s}$ are the Lagrangian reference coordinates of the structure, and $\chi (X,t)\in \Omega_{t}^{s}$ is the current position of material point ***X*** at time *t*. Thus, the region occupied by structure at time *t* is $\Omega_{t}^{s}=\chi (\Omega_{0}^{s},t)$, and $\Omega_{t}^{f}=\Omega \setminus \Omega_{t}^{s}$ is the region occupied by the fluid at time *t*.

The governing equations for the coupled fluid‐structure system are

(1) $$\;\rho \left(\frac{\partial u(x,t)}{\partial t}+u(x,t)\cdot \nabla u(x,t)\right)=-\nabla p(x,t)+\mu \nabla^{2}u(x,t)+f(x,t),$$

(2) ∇·u(x,t)=0,

(3) $$\;f(x,t)=\underset{\Omega_{0}^{s}}{\int }F(X,t)\;\delta (x-𝛘(X,t))\;dX,$$

(4) $$\;\underset{\Omega_{0}^{s}}{\int }F(X,t)\cdot V(X)\;dX=-\underset{\Omega_{0}^{s}}{\int }P^{e}(X,t):\nabla_{X}V(X)\;dX,\;\forall V(X),$$

(5) $$\;\frac{\partial \chi }{\partial t}(X,t)=u(\chi (X,t),t)=\underset{\Omega }{\int }u(x,t)\;\delta (\chi (X,t)-x)\;dx,$$

with ***V***(***X***) an arbitrary Lagrangian test function. Equation (1) is the momentum equation for the coupled system. *ρ* and *μ* are the material density and dynamic viscosity. *p*(***x***,*t*) and ***u***(***x***,*t*) are the material pressure and velocity. ***f***(***x***,*t*) represents the Eulerian force density in $\Omega_{t}^{s}$ that is induced by structure deformation. Equation (2) is the incompressibility constraint in Eulerian form. Equations (3) and (5) are interaction equations between the Lagrangian and Eulerian coordinates, and the three‐dimensional Dirac delta function *δ*(***x***) is used here to transfer data between the two frames. ***F***(***X***,*t*) represents the Lagrangian force density in the finite element space obtained by the projection of the first Piola‐Kirchhoff stress tensor $P^{e}(X,t)$ through (4). Equation (5) implies the no‐slip condition in the fluid‐structure interface.

### 2.2 Energy analysis

We focus on two types of energy change, the kinetic energy (KE)

(6) $$\text{KE}=\underset{\Omega^{\text{LA chamber}}}{\int }\frac{1}{2}\rho u\cdot u\;dx,$$

and the rate of energy dissipation (D)

(7) $$\;D=\int_{\Omega^{\text{LA chamber}}}\mu (\nabla u+\nabla u^{T}):\nabla u\;dx,$$

where ^LA chamber^ is the flow region inside the LA chamber.

### 2.3 Spatial and temporal discretization

We use a Cartesian grid to discretize the Eulerian domain with a staggered grid for the Eulerian velocity ***u***=(*u*_*x*_,*u*_*y*_,*u*_*z*_), pressure *p*(***x***,*t*), and force density ***f***=(*f*_*x*_,*f*_*y*_,*f*_*z*_). Finite difference approximations are used for the divergence, gradient, and Laplace operators with second‐order accuracy.^45^ The nonlinear advection terms in Equation (1) are discretized using a version of piecewise parabolic method (PPM).^46^ The Lagrangian domain is discretized using finite element approach with a trilinear basis function for the displacement and Lagrangian force density ***F***(***X***,*t*). For more details of the discretization, readers are referred to Griffith and Luo.^44^

## 3 MODEL GEOMETRY AND MATERIAL PROPERTIES

### 3.1 LA and MV geometry

The LA geometry used in the current study is based on one of recently published LA geometry dataset,^27^ which was collected from a 35‐year‐old male patient with hyperlipidemia who underwent a clinically indicated coronary computed tomography angiography (CTA).^27^ The coronary CTA images were first segmented in Seg3D 1
http://www.sci.utah.edu/cibc-software/seg3d.html
, then smoothed via a combined smoothing and upsampling algorithm to obtain sufficient spatial resolution for computational finite element mesh generation. The Octree‐based mesh generation software Tarantula (CAE Software Solutions, Eggenburg, Austria) was used to generate the tetrahedral LA mesh.

**Figure 1:**
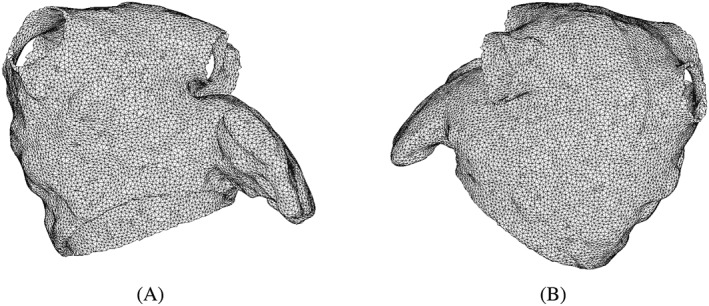
The finite element mesh of the LA model at (A) anterior view and (B) posterior view

In the original dataset,^27^ the total number of mesh elements is 121 207 799, and the total number of mesh nodes is 21 868 400 , which is overly fine for the purpose of our mechanical modelling. Therefore, the reduction of mesh density is needed and achieved via CGAL 2
https://www.cgal.org
 and TetGen (Weierstrass Institute for Applied Analysis and Stochastics, Berlin, Germany). Figure 1 shows the coarsened LA mesh with 92 590 elements and 28 808 nodes, following a grid‐independence test. Table 1 summarizes the geometric features for the LA geometry.

**Table 1:** LA geometry information

	LA Geometry
LA main chamber volume	107.3 mL
LA appendage volume	12.6 mL
Wall thickness	1.5 mm (average, non‐uniform)
RSPV orifice area	2.5 cm^2^
RIPV orifice area	2.0 cm^2^
LSPV orifice area	1.8 cm^2^
LIPV orifice area	2.5 cm^2^
Appendage orifice area	4.6 cm^2^

Abbreviations: LIPV, left inferior pulmonary vein; LSPV, left superior pulmonary vein; RIPV, right inferior pulmonary vein; RSPV, right superior pulmonary vein.

**Figure 2:**
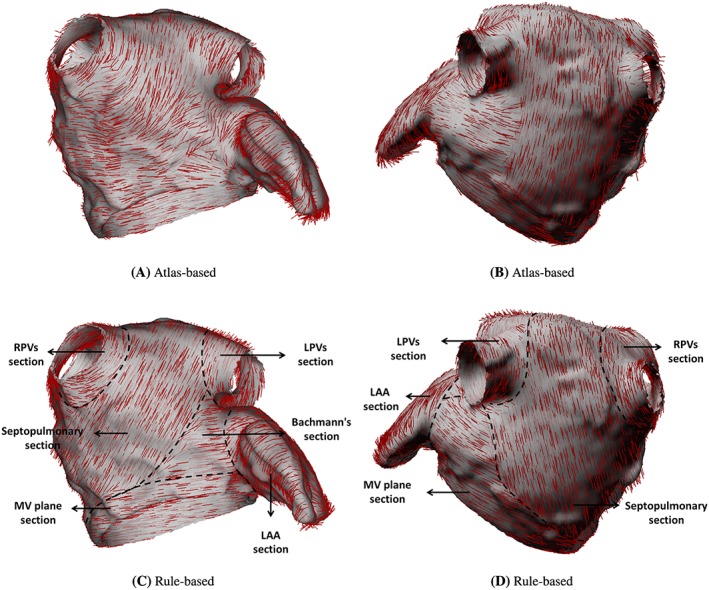
Fibre structures of the LA model from anterior (left) and posterior (right) views

The LA has complex fibre architecture composed of dominant muscle bundles with different orientations across the wall. Therefore, it requires significant amount of work to define a physiologically detailed fibre structure for the material model. In the current study, two fibre structures are compared. The first fibre structure is based on the work by Fastl et al,^47^ who used an atlas‐based method for the atrial fibre construction in their original dataset. In brief, an average atrial geometry, generated by the combination of 30 MRI datasets with 122 predefined landmarks, is constructed first and then used to transfer landmarks to the end‐ and epicardial surface on personalized atrial geometry. Next, 272 auxiliary lines were computed by the landmarks and subdivide atrial surface into 151 atrial regions in which the surface fibre orientations were determined. Finally, the fibre orientations for tetrahedral elements were computed via transmural interpolation. Following the aforementioned mesh density reduction, the fibre orientations are directly mapped from the original dataset to the coarsened LA mesh by a nearest neighbour approach, shown in Figure 2A,B.

The second fibre structure is defined using a rule‐based method similar to the study of Ferrer et al^47^ but with fewer atrial regions. The LA is divided into six sections as shown in Figure 2C,D, and described below:

- the left pulmonary veins (LPVs) section: circumferentially distributed fibres around the LSPV and LIPV;
- the right pulmonary veins (RPVs) section: circumferentially distributed fibres around RSPV and RIPV;
- the septopulmonary section: fibre structures mainly included in the septopulmonary muscle bundle which arises from the anterosuperior septal raphe, runs obliquely to superior wall and then back to atrial septum;
- the bachmann's section: circumferentially distributed fibres on anterior wall, and gradually blending into the circular fibres parallel to the MV annulus.
- the LAA section: circumferentially distributed fibres around the appendage central axis; and
- the MV plane section: circumferentially distributed fibres parallel to the MV annulus plane.

It should be mentioned that the current rule‐based method does not include transmural fibre change. However, it captures the major characteristics of LA muscle bundles. For example, at the epicardial aspect, the behaviour of Bachmann's bundle as well as the septopulmonary bundle are included in septopulmonary and Bachmann's sections. At the endocardial aspect, even though the septoatrial bundle, which originates from the anterior interatrial raphe and blends with the septopulmonary bundle on the LA superior roof and circumferential fibres on the lateral and posterior walls, is not explicitly defined, the major pattern is included in septopulmonary and MV plane section. More details of the atrial fibre morphology and the ruled‐based method are provided in Appendix A.

**Figure 3:**
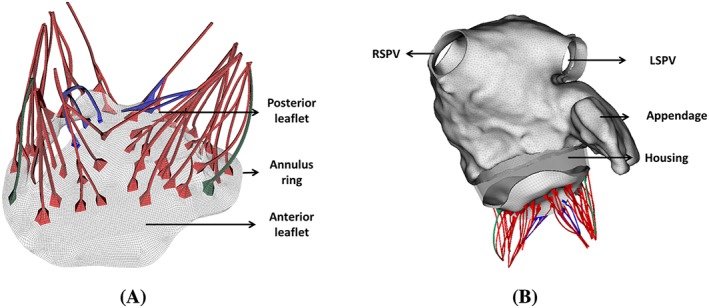
MV geometry (left) and coupled LA‐MV model with a rigid housing (right). Strut chordae are colored in red, marginal chordae are colored in blue, and basal chordae are colored in green

The MV model is based on the work by Wang et al,^48^ and the MV geometry is reconstructed from multi‐slice computed tomography (MSCT) scans of a normal mitral valve at mid‐diastole from a 61‐year‐old male patient. It contains detailed leaflets, chordae geometry, and fibre structure, shown in Figure 3A. Details of the MV model can be found in our previous work.^43^, ^48^ A rigid housing structure is then used to connect the LA and MV geometries as shown in Figure 3B. Further details of the LA and MV model connection are provided in Appendix B.

### 3.2 Material properties

Constitutive models for the MV model and their parameter values are taken to be the same as in our previous work.^43^ To model the passive material response of LA wall, we use an invariant‐based, transversely isotropic constitutive model

(8) $$W^{\text{LA‐passive}}=\frac{a}{2b}\{\exp[b(I_{1}-3)]-1\}+\frac{a_{1}}{2b_{1}}\{\exp[b_{1}{\left(I_{4}-1\right)}^{2}]-1\}+\frac{\beta }{4}\log^{2}(I_{3}),$$

in which $I_{1}=\text{tr}(F^{T}F)$, $I_{3}=\det(F^{T}F)=J^{2}$, with F=∂𝛘/∂X being the deformation gradient and J=det(F). Fibre deformations are described through $I_{4}=e\cdot (F^{T}F)e$, and the fibre orientation ***e*** at the reference state is shown in Figure 2. In particular, the fibres are defined only to support extension, so compressed fibres do not contribute to the strain energy function. The last term in the energy, $\frac{\beta }{4}\log^{2}(I_{3})$, acts to penalize compressible deformations.^49^ We choose *β*=500 kPa. *a*=8.0 kPa, *b*=5.57, *a*_1_=6.0 kPa, and *b*_1_=4.06. Due to the lack of experimental data, these parameters have been tuned, based on the ventricular myocardium,^50^ to produce physiologically realistic cardiac output in the LA.

Similar to the previous approach,^51^ we define the modified elastic stress tensor $P^{e}$ via

(9) $$\begin{array}{ccc} P_{\text{LA‐passive}}^{e}= & \;a\exp[b(I_{1}-3)]F-a\exp[b(I_{1}-3)]F^{-T} & \\ & +2a_{1}(I_{4}-1)\exp[b_{1}{\left(I_{4}-1\right)}^{2}]Fe⊗e\\ & +\beta \log(I_{3})F^{-T}. \end{array}$$

The term $a\exp[b(I_{1}-3)]F^{-T}$ is included to ensure $P_{\text{LA}}^{e}$ is zero when F=I.

**Figure 4:**
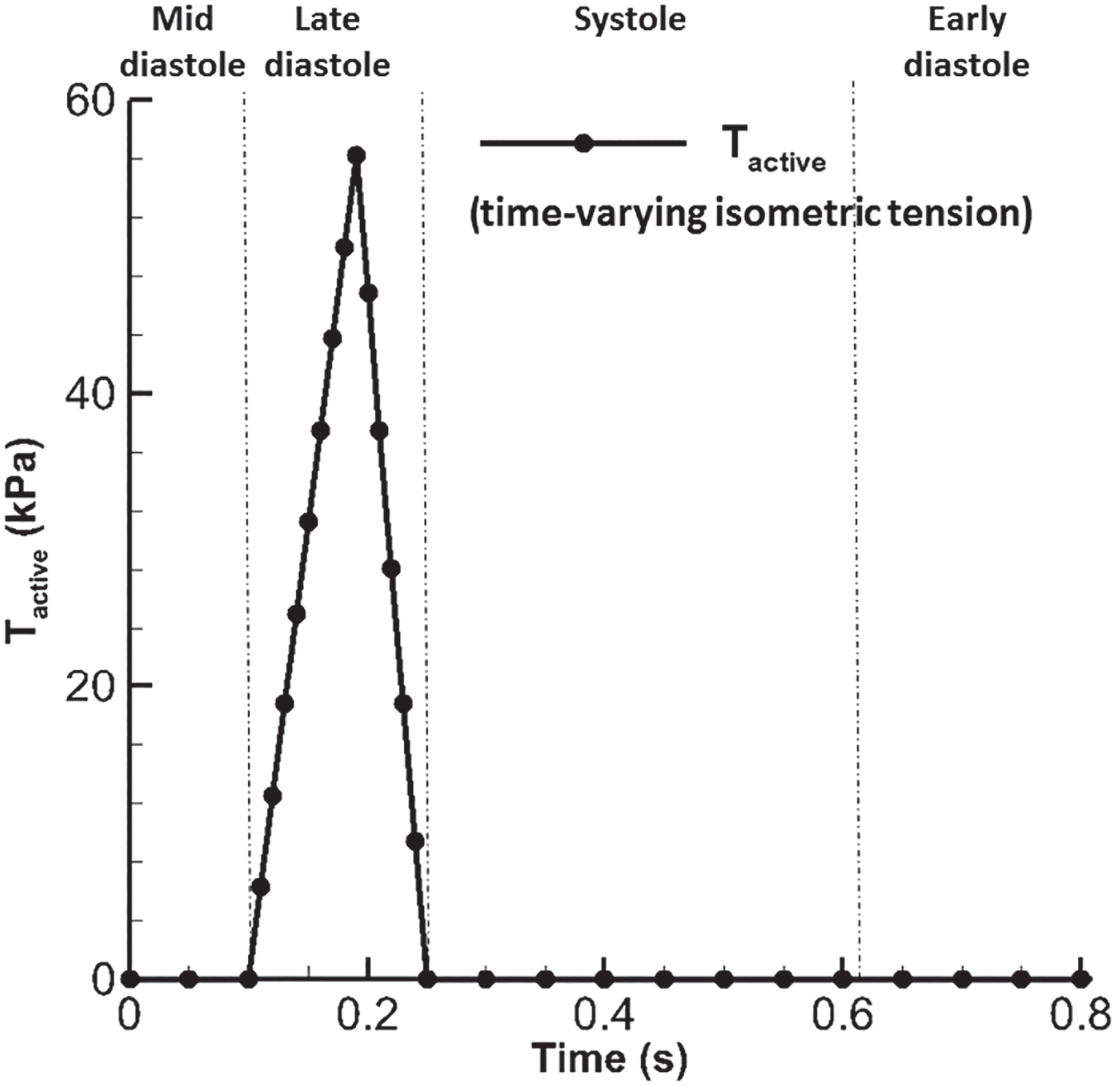
The profile of T_active_ with a maximum value^52^ of 56.2 kPa

To account for the active contraction at late diastole initiated by the LA wall, we add a simplified active stress tensor to the overall elastic stress tensor similar to Wang et al,^53^

(10) $$P_{\text{LA‐active}}^{e}=JTFe⊗e,$$

in which

(11) $$T=T_{\text{active}}[1+\gamma (\lambda -1)],$$

where _active_ is the time‐varying isometric tension as shown in Figure 4, *γ* = 4.9 from Niederer et al,^52^ and $\lambda =\sqrt{I_{4}}$ is the fibre stretch ratio.

## 4 DYNAMIC LA‐MV MODEL IMPLEMENTATION

**Figure 5:**
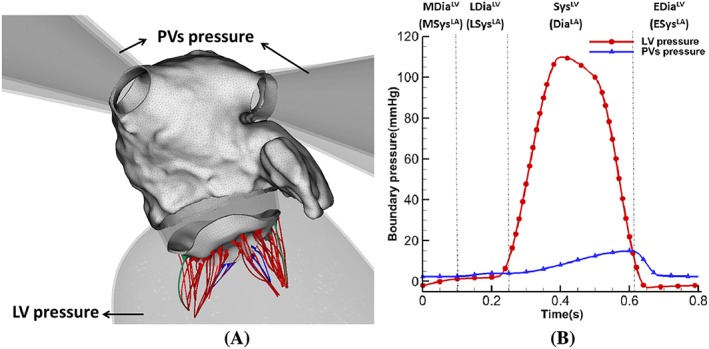
(A) Coupled LA‐MV model mounted on rigid PVs and LV tube. (B) The applied pressure profiles with four phases: systole (Sys^LV^), early diastole (EDia^LV^), mid diastole (MDia^LV^), and late diastole (LDia^LV^) based on left ventricular dynamics. Or diastole (Dia^LA^), early systole (ESys^LA^), mid systole (MSys^LA^), and late systole (LSys^LA^) based on left atrial dynamics

The coupled LA‐MV model is immersed in a viscous fluid with density 1 g·cm^−3^ and dynamic viscosity 0.04 g·cm^−1^·s^−1^. The computational domain has the size of 23.6 cm × 21.0 cm × 20.8 cm. Four PVs and the LV are represented by rigid tubes to mount the LA‐MV model on the domain boundaries where pressure boundary conditions are applied as shown in Figure 5. The coupled LA‐MV system undergoes a 0.1‐second initialization phase followed by several cardiac cycles. After the second cycle, the results are converged to a steady stage. Hence, the results from the second cardiac cycle are presented.

In the current MV model, there are 10 chordal origins representing the locations at papillary muscles. Similar to the work by Wang et al,^48^ each origin is applied with a time‐dependent displacement boundary condition to mimic the movement of papillary muscle, which is based on the CT scans at mid diastole and mid systole. The MV annulus, however, remains fixed during the simulation. For the LA geometries, the structural mesh element size is around 0.1 cm for the edge length. For the fluid mesh, two‐level block‐structured adaptively refined grid with a refinement ratio 4 between levels is used, and the mesh size is 0.13 cm × 0.13 cm × 0.13 cm. An explicit scheme is used to solve the IB/FE system, and a small time step size of 0.01 ms is chosen to ensure stability and numerical convergence.

To study various factors affecting LA biomechanical function, we consider the following six cases:

1. The normal case, denoted as (LA^Original^) : parameters and boundary conditions for the LA and the MV are adjusted to produce normal LA cardiac output. The LA contains atlas‐based fibre structure.
2. The atrial fibrillation (AF) case (LA^AF^): derived from LA^Original^ without active contraction by setting T_active_=0, and the MV functions normally. This is to mimic the extreme situation of atrial fibrillation when the LA wall can not contract at all and used to investigate the effect of a lack of atrial kick at end‐diastole in the case of atrial fibrillation.^24^ The occurrence of high‐frequency fibrillation of atrial wall is not included here.
3. The mitral valve regurgitation (MVR) case (LA^MVR^): derived from LA^Original^ by moving the chordae origins towards LA to cause MV leaflets collapse and regurgitant flow during systole. This case will be used to study the influences of MV regurgitation on the LA function.
4. The rule‐based fibre case (LA_S_^RB^): derived from LA^Original^, the only difference is that the fibre structure is generated using the rule‐based method. This case will allow us to investigate the impact of different fibre structures on LA dynamics.
5. The uniform wall thickness case (LA_S_^1.5^): derived from LA^Original^, but with a uniform wall thickness of 1.5 mm which is the averaged wall thickness of the original LA geometry, others are kept the same as LA^Original^. This will allow us to study the effect of wall thickness uniformity on LA dynamics.
6. The thickened wall case (LA_S_^2.2^): also derived from LA^Original^ but with a uniform wall thickness of 2.2 mm, about 50% thicker than LA_S_^1.5^ which could represent LA wall thickening in the case of atrial fibrillation.^54^

The IB/FE framework used in the current study is implemented in the open‐source IBAMR software 3
https://ibamr.github.io
. IBAMR is an adaptive and distributed‐memory parallel implementation of the immersed boundary method leveraging several open‐source computational frameworks, including SAMRAI 4
https://computation.llnl.gov/projects/samrai
, PETSc 5
https://www.mcs.anl.gov/petsc
, libMesh 6
http://libmesh.github.io
, and *hypre* 7
http://www.llnl.gov/casc/hypre
to perform core functionality. All simulations are performed at the School of Mathematics and Statistics at the University of Glasgow using Linux servers with Intel Xeon E5‐2660 v3 2.60 GHz CPUs and 128 GB RAM. A typical simulation of one cardiac cycle using 28 processors takes about 9 days in wall‐clock time.

## 5 RESULTS

### 5.1 Normal and pathological LA models

In this section, LA functions under pathological conditions are analysed among LA^Original^, LA^AF^, and LA^MVR^. Table 2 summarizes the stroke volume, the conduit volume, LA emptying volume, and the MV regurgitant volume in one cardiac cycle, where the stroke volume is obtained via the sum of the other three volumes. LA^Original^ has the largest stroke volume and conduit volume, which are in the healthy range (stroke volume: 95 mL ± 14 mL^55^; conduit volume: 42.6 mL ± 14.6 mL^56^). Compared with LA^Original^, LA^AF^ has smaller stroke volume with increased conduit volume and much reduced LA emptying volume, which can be explained by the absence of active contraction in late‐diastole. However, for LA^MVR^, it has much higher regurgitation volume due to the prolapse of the MV leaflets, which also leads to a much larger LA emptying volume during systole than LA^Original^.

**Table 2:** Cardiac output in models

	Stroke Volume, mL	Conduit Volume, mL	LA Emptying Volume, mL	Regurgitant Volume, mL
LA^Original^	98.5	46.5	58.0	6.0
LA^AF^	85.1	64.1	28.1	7.1
LA^MVR^	91.5	47.9	81.7	38.1

**Figure 6:**
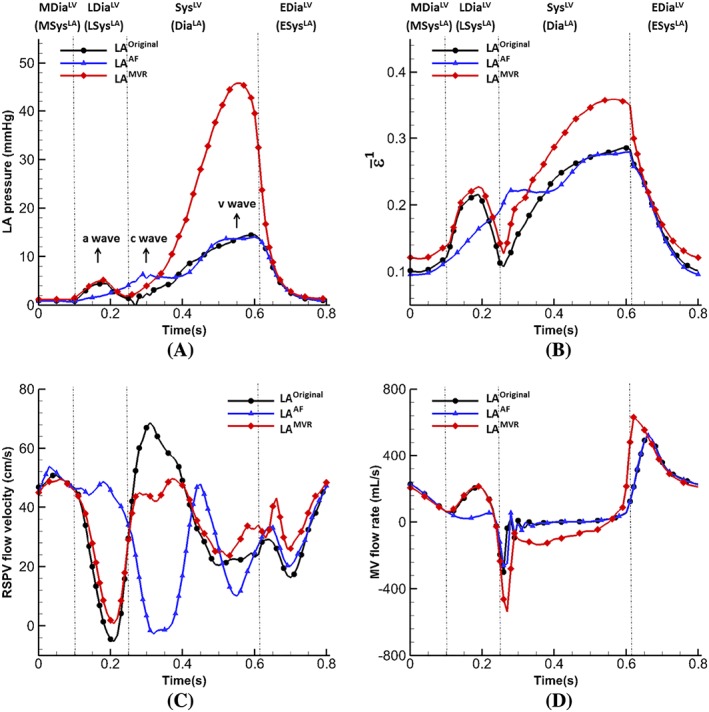
Comparison of results of the normal, AF and MVR cases: (A) pressure inside the LA main chamber, and (B) the averaged maximum principal strain ${\bar{\varepsilon }}^{1}$, (C) Flow velocity at right superior pulmonary vein orifice, and (D) Flow velocity at MV leaflet free edges during diastole

Figure 6A,B shows the pressure inside the LA and averaged maximum principal strain ( ${\bar{\varepsilon }}^{1}$) at the LA wall. During systole, a sharp peak (known as the v‐wave) appears in the LA pressure for LA^MVR^ because of the regurgitant flow. However, the pressure in LA^AF^ has a pronounced early systolic wave (known as the c‐wave, resulting from MV closure). The peak of the v‐wave is 14.5 mmHg (LA^original^) and 14.1 mmHg (LA^AF^), much lower than in LA^MVR^ (46.0 mmHg). In diastole, LA^MVR^ appears to be in line with the normal case, but the a‐wave (caused by the atrial contraction at end diastole) has disappeared in LA^AF^ because it lacks active contraction. Similar trends are seen in the maximum principal strain curves, a much higher strain value can be found in LA^MVR^ in systole compared with the other two cases.

Figure 6 C is the mean blood velocity profile at RSPV. In general, RSPV velocities are similar for both the normal and MVR cases, except at early‐systole due to the large MV regurgitation into the LA cavity, which restricts the filling flow from RSPV in the MVR case. More substantial difference in the RSPV flow is seen in the AF case, especially from mid‐diastole to end‐systole. No regurgitation occurs in the AF case at RSPV at late‐diastole, and the filling in early‐systole is much smaller because the LA has already been passively stretched, as shown in Figure 6B. Figure 6D is the flow near the MV leaflet free edges for the three cases, again, large difference for the AF case can be found in late‐diastole compared to the normal case, and a very high regurgitant flow during systole in the MVR case as expected.

**Figure 7:**
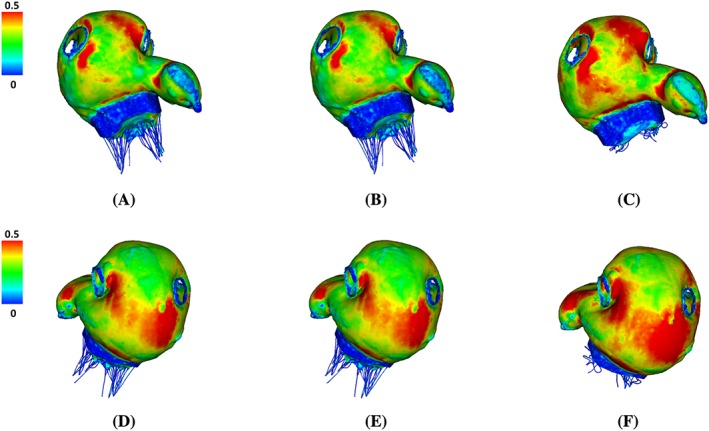
Distribution of the maximum principal strain (*ε* ^1^) at end‐systole (*t*=0.61 s) for LA^Original^ (A,D), LA^AF^ (B,E) , and LA^MVR^ (C,F). Top row: anterior view. Bottom row: posterior view

**Figure 8:**
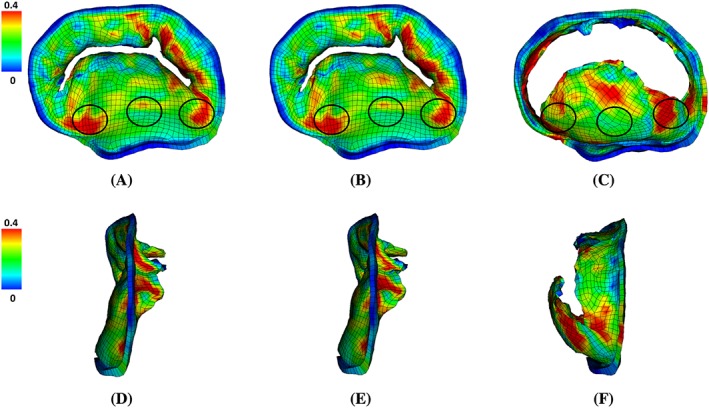
The maximum principal strain distributions on the MV at mid‐systole (*t*=0.4 s) in (A,D) LA^Original^, (B,E) LA^AF^ and (C,F) LA^MVR^. Top row: front view (from atrium side). Bottom row: side view (atrium to the left). The belly of anterior leaflet and the two trigon areas with higher strains are circled

Figure 7 shows the distribution of the maximum principle strain *ε* ^1^ on the LA wall at end‐systole. All three cases have similar strain patterns, but the MVR case has much higher strain level. It is also associated with a substantial enlarged LA cavity (end‐systole cavity: 187.2 mL (LA^Original^) vs 186.6 mL (LA^AF^) vs 204.5 mL (LA^MVR^)). Figure 8 shows the distribution of (*ε* ^1^) on the MV itself at mid‐systole. A small gap can be found for both the normal and AF cases. In the present immersed boundary formulation, the gap will remain when the valve is closed and loaded because of the regularized delta function in the Lagrangian‐Eulerian coupling operators. The size of this gap is determined by the amount of regularization, which in the present methodology is tied to the background Eulerian grid spacing. Despite this gap, the valve is hydraulically closed, in that it is able to support an adverse pressure difference without leak. And both cases have similar strain patterns, ie, higher at the two trigon areas of the anterior leaflet and the commissure area of the posterior leaflet. This observation agrees with previous studies.^43^, ^59^, ^60^, ^61^ However, in the MVR case, both leaflets collapse towards the atrium side as shown in Figure 8C,F, and *ε* ^1^ at the trigons (average value) are higher than other two cases as listed in Table 3.

**Table 3:** Maximum principal strain (averaged) on MV anterior leaflet together with published experimental data

Case	Anterior Belly	Trigon (Left)	Trigon (Right)
LA^Original^	0.21	0.29	0.28
LA^AF^	0.20	0.29	0.28
LA^MVR^	0.19	0.32	0.27
Jimenez et al^57^	0.22 ± 0.07^*r*^		
Sacks et al^58^	0.16 ± 0.20^*r*^		

*Note*. The superscript *r* indicates the radial direction.

**Figure 9:**
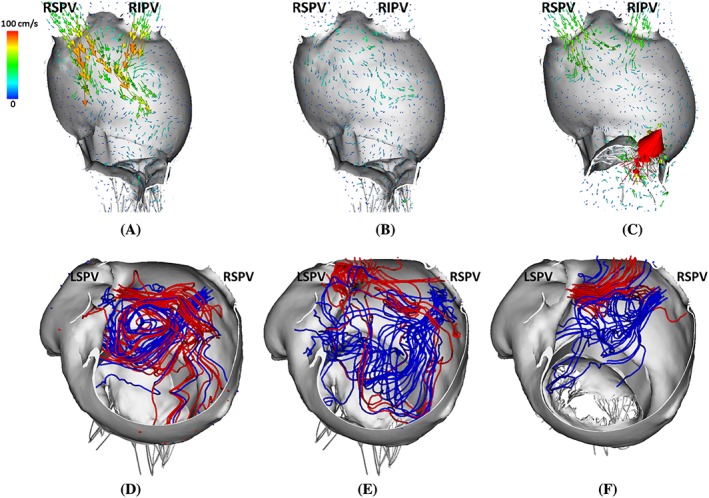
Upper panel: side view of flow fields inside LA at early systole (*t*=0.35 s) for LA^Original^ (A), LA^AF^ (B), and LA^MVR^ (C). Lower panel: top view of streamlines with seeds near LSPV (red) and RSPV (blue) at early‐systole (*t*=0.35 s) for LA^Original^ (D), LA^AF^ (E), and LA^MVR^ (F)

Figure 9A‐C shows flow vectors inside the LA at early‐systole. High filling flows at the pulmonary veins can be found in the normal case and to a lesser degree in the MVR case. No clear filling pattern is shown in the AF case because of the elevated LA pressure at late‐diastole due to the lack of active emptying process. As expected, a regurgitant jet towards the LA posterior wall can be found in the MVR case as shown in Figure 9C, which substantially affected the filling process. Figure 9D‐F shows the streamlines at early‐systole starting from the LSPV and RSPV orifices. A clear clockwise vortex inside the LA main chamber is seen in the normal case from the LA posterior view. However, when regurgitation occurs, due to the disturbance caused by the regurgitated jet, the clockwise vortex is very weak (Figure 9F).

**Figure 10:**
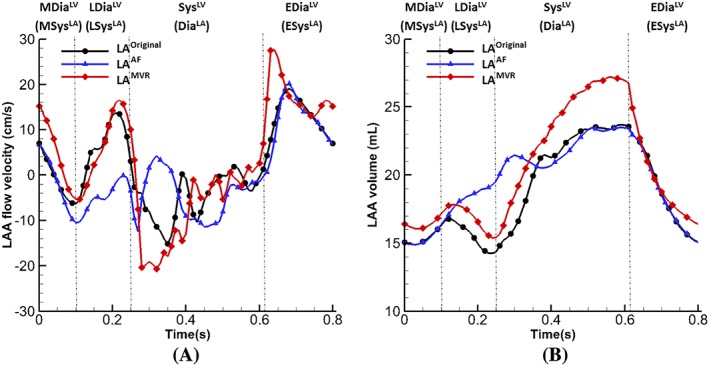
(A) Flow velocities at the left atrium appendage orifice and (B) left atrium appendage volume from cases of LA^Original^, LA^AF^ and LA^MVR^

More quantitative information is given in Figure 10A, which plots the flow velocity at the LAA orifice during one cardiac cycle. During early diastole, there is an emptying flow wave in all three cases, followed by a late‐diastole peak resulted from the LAA active contraction for the normal and MVR cases but not in the AF case. Consequently at the beginning of systole, a large filling wave only appears in the normal and MVR cases (with peak values −15.1 cm/s and −20.7 cm/s, respectively).

Figure 10B shows the LA appendage volume change in the three cases. There is no volume reduction at late‐diastole in the AF case and a substantial enlargement during systole in the MVR case. In order to quantitatively measure the effect of the LAA flow velocity to the flow inside LAA, we calculate the LAA fluid residence time (FRT) as follows: First, the fluid pathlines are generated with seeds at the LAA orifice, starting from the early ventricular systole when the first LAA filling wave occurs and ending at mid diastole when the first LAA emptying wave finishes. Then, the fluid particles that manage to exit from LAA are selected and the average time they stay inside the LAA is defined as the FRT.

The FRT in the MVR case (0.394 s) is less than that in the normal case (0.423 s), and the AF case has the largest FRT value (0.470 s) indicating that blood entering the LAA during systole stays much longer in the LAA compared with other two cases.

**Figure 11:**
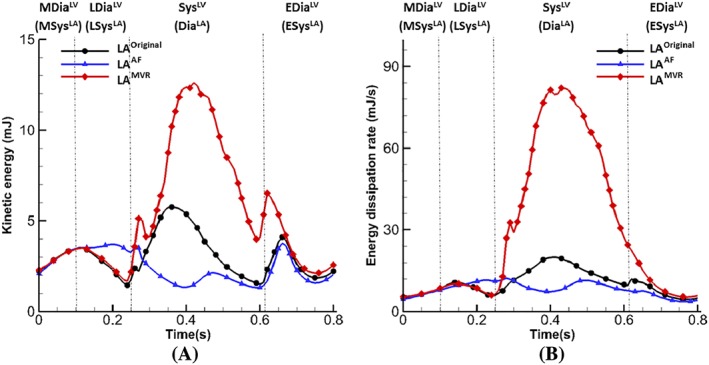
Energy budget analysis. (A) Kinetic energy of flow inside the LA and (B) energy dissipation rate of flow, integrated within the LA

The performance of the pathological cases can be better represented by the energy budget analysis. Figure 11 plots the total flow kinetic energy (KE) and dissipation rates (D) inside the LA. The MVR case has the highest kinetic energy and dissipation rates at systole compared with other two cases, which suggests the after‐load of the LV is increased and a part of the LV work is used to overcome this rather than pumping the blood. On the contrary, the AF case has the lowest levels of kinetic energy and dissipation rate without peaks at systole, indicating an extremely inefficient filling function.

In summary, in the absence of atrial kick at end‐diastole as in LA^AF^, the filling process is greatly impaired (stroke volume reduced 15.8%). As a result, the LA pressure at early‐systole is elevated, the PV filling flows are suppressed, and the blood residential time is longer than the normal case (0.470 s vs 0.424 s) which may suggest a higher risk of blood stagnation (77.6% vs 27.5% of LAA region with flow velocity less than 10  cm/s at early‐systole), hence thrombus formation. In the case of MV regurgitation as in LA^MVR^, the LA cavity is substantially enlarged with much increased LA pressure and the first principle strain during systole. The MV regurgitant jet further disturbs the PV filling flow inside LA, which also results in a higher level of energy dissipation compared to the normal case LA^Original^. However, the regurgitant jet flow in LA^MVR^ seems to help wash out the residual fluid inside the LAA during systole.

### 5.2 Effects of modelling variations

**Figure 12:**
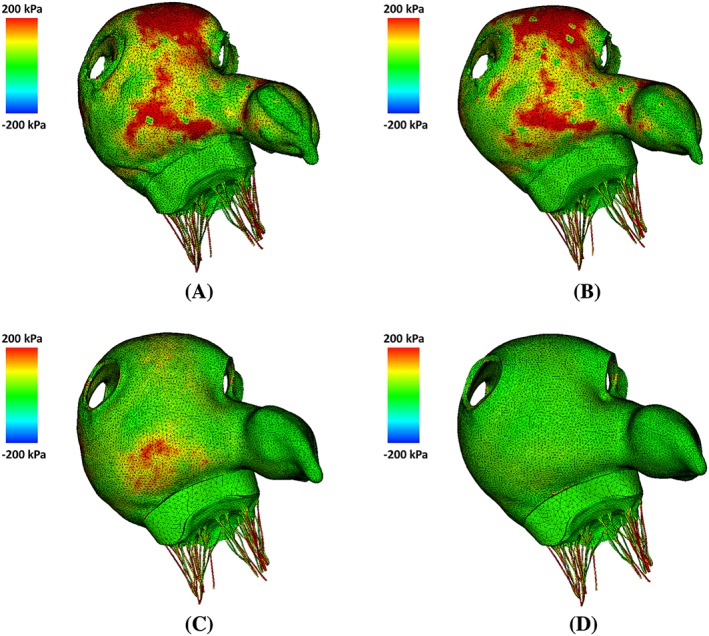
The maximum principal stress distributions at end‐systole (*t*=0.61 s) for (A) LA^Original^, (B) LA_S_^RB^, (C) LA_S_^1.5^, and (D) LA_S_^2.2^ in the anterior view

In this section, we compare the LA function with varied features, including atlas‐based (LA^Original^) vs rule‐based fibre structures (LA_s_^RB^), and non‐uniform (LA^Original^) vs different uniform wall thickness (1.5 mm—LA_s_^1.5^, or 2.2 mm—LA_s_^2.2^). Figure 12 shows the maximum principal stress distributions on the LA wall at end‐systole for these different modelling variations. Figure 12A,B shows that the varied fibre‐structure has a moderate effect on the LA stress distribution. However, the impact of the uniform thickness is more substantial. The stress in LA_s_^1.5^ (Figure 12C) is much lower compared with the original LA model (LA^Original^) even though the uniform thickness is equivalent to the average thickness of LA^Original^. With further increased wall thickness in LA_s_^2.2^, the maximum principal stress has the lowest levels as shown in Figure 12D.

**Figure 13:**
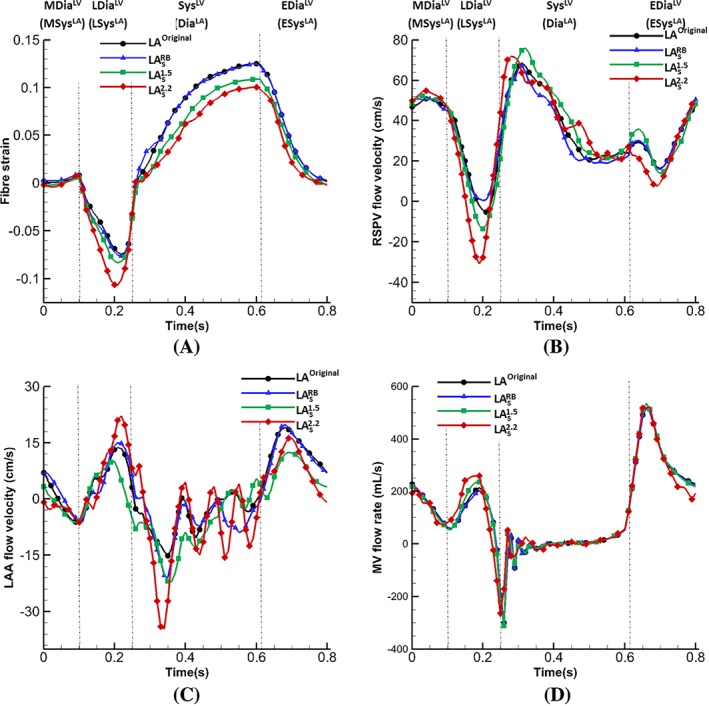
Comparisons of average atrial fibre strain (A), and flow velocity at the (B) right superior pulmonary vein orifice, (C) left atrium appendage orifice, and (D) the flow rate across the MV

The information revealed in Figure 12 is more clearly presented in Figure 13. Figure 13A plots the overall averaged fibre strain on atrial walls. The models with uniform wall thickness markedly underestimate the value of fibre strain compared to LA^Original^. Figure 13B is the venous flow velocity at the RSPV orifice, LA_s_^2.2^ has the largest regurgitation at late‐diastole (*t*=0.19 s) and higher flow velocity at the LAA orifice at late‐diastole (*t*=0.21 s) and early‐systole (*t*=0.33 s) as shown in Figure 13C. The flow rates at the MV orifice are similar, although the one for LA_s_^2.2^ is slightly higher (Figure 13D) at late‐diastole. The FRTs inside the LAA are similar in all three cases LA_s_^RB^ (0.402 s), LA_s_^1.5^ (0.405 s), and LA_s_^2.2^ (0.408 s).

## 6 DISCUSSION

This paper presents a FSI model of left atrium with physiologically realistic geometry and fibre structure, combined with a previously developed mitral valve model with detailed leaflets and chordae tendineae.^43^ The combined model is used to study both the structure mechanical behaviour and haemodynamics under different conditions. Compared with previous LA modelling work, several advances have been made in the current study: (a) a realistic MV model is coupled to the LA, and this is the first time that such a model is included in LA modelling and analysed within a FSI framework; (b) effects of atrial fibrillation and MV regurgitation on overall LA performance are analysed in detail; and (c) effects of wall thickness and fibre structures on LA dynamics are studied.

As a closely related structure to LA, MV plays an important role in the LA function. In the current work, we show that with a three‐dimensional MV model, more realistic interaction between LA and MV can be captured, especially in the case of MV dysfunction. For example, because of the regurgitation in systole, LA^MVR^ suffers an excessive enlargement in LA cavity during systole with significantly higher pressure, shown in Figure 6A,B, compared with the cases with complete MV closure. Similar findings are also reported by Kihara et al,^62^ in which LA experiences enlargement in size and mass as well as increased mean pressure in the case of mitral regurgitation. Furthermore, the flow vortex seen in normal LA function (Figure 9) disappears because of the strong regurgitant jet. And the flow rate cross the MV during diastole also shows different behaviours in the case of AF and MVR, shown in Figure 6D, which could potentially have strong impact on the LV pump function. Because the model uses a rigid housing for LA‐MV connection, the simulations do not account for the dynamics of the MV annulus, including its shape change and longitudinal movement. We plan to study the impact of annular dynamics on the transmitral flow patterns and overall energetics in future work.

Previously, the LA wall‐blood interaction had not been studied in detail: a common practice in literature is to prescribe the wall motion with a focus on LA haemodynamics .^23^, ^24^, ^25^, ^26^ In our model, a full FSI analysis is performed using an immersed boundary method with finite element elasticity.^43^, ^44^, ^51^, ^60^, ^63^ Incorporating nonlinear hyperelastic anisotropic material models to the immersed boundary framework we are able to investigate how wall thickness and fibre structure affect LA function. Our results suggest that from a haemodynamics perspective, the simplified rule‐based fibre structure without transmural fibre orientation provides a good approximation for the detailed atlas‐based fibre structure with transmural fibre orientation, because both lead to similar LA flow pattern and wall deformation shown in Figures 12 and 13. Therefore, such simplified atrial fibre defining method may be used in the fluid‐structure interaction modelling of LA to reduce the model complexity. However, it has to be pointed out that we have not considered electrophysiology (EP) in the current model, and previous work has suggested the LA function can be more sensitive to fibre structure when including EP.^47^, ^64^ Another key geometric factor in the LA modelling is the wall thickness. LA has non‐uniform wall thickness^1^ where the posterior wall and superior wall are in general thicker than other regions. As detailed LA geometry construction requires high quality medical images, some models are based on uniform wall thickness.^33^, ^34^ Our results show that non‐uniform and uniform wall thickness lead to quite different results in mechanical behaviours, for example, the LA model with uniformly wall thickness (LA_s_^1.5^) is less stretched throughout the cardiac cycle shown in Figure 13A. Therefore, if realistic LA wall dynamics are required, caution must be taken when applying such simplification during the geometry reconstruction process. Furthermore, with an increase in wall thickness by approximately 50%, our LA‐MV model exhibits higher LAA flow velocities (Figure 13C), larger reversal PV flow (Figure 13B), and late diastolic MV flow (Figure 13D) compared with LA^Original^, which suggests the substantial impact of atrial structural remodelling on the flow. Although the current model uses a relatively simple approach to mimic the remodelling process by directly increasing wall thickness, it shows the potential to capture more detailed mechanics and flow changes when combined with a physiologically realistic growth and remodelling system.

The left atrial appendage also plays a key role in the LA pump function. Previous studies have shown that it is a most common site for thrombus formation in the case of atrial fibrillation.^65^, ^66^, ^67^ As a key indicator of LAA thromboembolic event, LAA flow velocity is closely related to the qualitative parameters of elevated thromboembolic risk. Kamp et al^68^ performed transoesophageal echocardiography in 88 patients with paroxysmal or chronic atrial fibrillation and found that there is a significant higher risk of thrombus formation in patients with the presence of spontaneous echo contrast in left atrium and particularly low left atrial emptying peak flow velocity (≤20 cm/s). Verhorst et al^69^ also discovered reduced LAA flow velocities among the patients with documented systemic embolism (emptying flow peak: 25 ± 19 cm/s; filling flow peak: 23 ± 15 cm/s) compared with those without systemic embolism (emptying flow peak: 39 ± 23 cm/s; filling flow peak: 33 ± 16 cm/s). By excluding the active contraction in LA^AF^, our results (Figure 10A) show that LAA active emptying wave disappears at end‐diastole compared to the normal case (peak: 13.7  cm/s) and the early‐systole filling wave has a reduced peak (11.4  cm/s (LA^AF^) vs 15.1  cm/s (LA^Original^)). The fluid residence time inside LAA is also much longer in the case of AF (0.470 s) compared with other cases (LA^Original^: 0.423 s; LA^MVR^: 0.394 s) during contraction, which could induce a higher risk of blood clot formation. In the case of MV regurgitation, our results (Figure 10A) show that a higher and earlier systole filling wave occurs, as a result, the fluid residence time in the LAA is reduced, and thus indicates a lower chance of thombus formation inside LAA This is consistent with the work by Kranidis et al,^38^ who found that significant MVR actually protects patients against LA thrombogenesis formation and systemic embolization when the patients have both MV and AF diseases. Karatasakis et al^70^ also found that the presence of significant MVR correlates with lower risks of thrombi and embolization.

The work by Arvidsson et al^71^ used cardiac magnetic resonance to quantify the atrial blood kinetic energy and three energy peaks were found at systole, early‐diastole, and late‐diastole.Our coupled LA‐MV model presents a similar kinetic energy pattern with three peaks, but the highest peak appears during systole instead of early‐diastole, as shown in Figure 11A. Also, the energy dissipation rate (Figure 11B) shows similar behaviour as the study of Wang et al,^72^ which found the three peaks at systole (the highest), early‐diastole, and late‐diastole. The LA dysfunction is also reflected by the change of energy field in LA^AF^ and LA^MVR^. For instance, both kinetic energy and energy loss (Figure 11) have much higher magnitude in the case of MVR than the normal case, while in the case of AF, on the contrary, lower energy level is seen due to the overall slower flow inside the LA. Such distinct energy behaviour among pathological cases implies that energy analysis provides a useful tool for the evaluation of cardiac function efficiency such as LA, LV pump function assessment.

Although there are no direct measurements available to compare our model predictions, our results are consistent with clinical observations. For example, De Marchi et al^73^ reported the peak systole flow velocity is 57(±18) cm/s and peak diastole flow velocity 49(±17) cm/s at RSPV among 315 normal patients. The peak flow velocities at RSPV from LA^Original^ are 68.7 cm/s in systole and 50.8 cm/s in diastole, which are in the range of the reported values from De Marchi et al.^73^In the work by Dahl et al,^74^ two transmitral mass flow peaks are observed using in vivo MRI measurements from a healthy male (early‐diastole peak: 480 mL/s approximately; late‐diastole peak: 200 mL/s approximately ) and the volume change of atrial cavity during diastole is about 50 mL calculated from segmentation data. In our result from LA^Original^, the transmitral flow rate also obtains two diastolic waves with early‐diastole peak 520.7 mL/s and late‐diastole peak 212.7 mL/s (Figure 6D). The LA emptying volume is 58.0 mL close to their calculation. Also, Chao et al^75^ found that most patients with chronic AF had early‐systole reversal flow at PV orifice and none had late‐diastole reversal flow, similar results can be found in LA^AF^ (Figure 6C). In a recent study, Ikenaga et al^76^ investigated the PV flow among patients before and after the MitraClip procedure, and they observed the immediate increase of systole PV flow in response to mitral regurgitation reduction, which is reflected in LA^MVR^ (Figure 6C). Nevertheless, this study has limitations, including (a) material properties for LA wall and mitral valve are not personalized because of lack of experimental data and (b) the connection of the LA and MV geometries uses a rigid housing structure, which leads to the fixed mitral annulus. In addition to the need of including MV annulus shape change during the cardiac cycle,^77^, ^78^ patient‐specific LV geometry is also required to provide more realistic structural boundary conditions for the LA‐MV model. In addition, prestrain is not included in the current material model for MV leaflets, but studies have shown the existence of substantial in vivo residual strains/stresses in the leaflets.^79^ Accounting for prestrain reduces the discrepancy between in vivo and ex vivo experimental measurements^80^ and assists with physiological deformations of MV at peak systole.^61^

## 7 CONCLUSIONS

In this study, we have developed a coupled LA‐MV model with fluid‐structure interaction implemented in an immersed boundary method with finite element hyperelasticity. The coupled LA‐MV model includes a detailed description of the atrial geometry, mitral valve leaflets, and chordae structure with fibre‐reinforced hyperelastic constitutive laws. LA‐MV dynamics is investigated under physiological and pathological conditions, including diminished LA active contraction and mitral regurgitation. We further study the effects of different geometric features on the coupled LA‐MV dynamics. Our results show that the lack of atrial contraction at late‐diastole leads to the absence of LA active emptying process and a high risk of blood stagnation in LAA. Compared with the normal case, the mitral regurgitation will result in higher LA pressure, enlarged LA cavity with higher energy dissipation caused by the disturbance of regurgitant jet but reduced fluid residence time in LAA, which is consistent with clinical observations. A rule‐based fibre structure can be a good approximation of the physiological fibre structure as long as the LA mechanics is of concern. However, LA wall thickness can play an important role in LA dynamics. For example, a uniform approximation of LA wall thickness can lead to under‐estimation of LA wall strain, and the thicker the wall, the lower the strain level. Our results suggest that care needs to be taken when reconstructing the LA geometry for patient‐specific modelling.

## Figures and Tables

**Figure A1:**
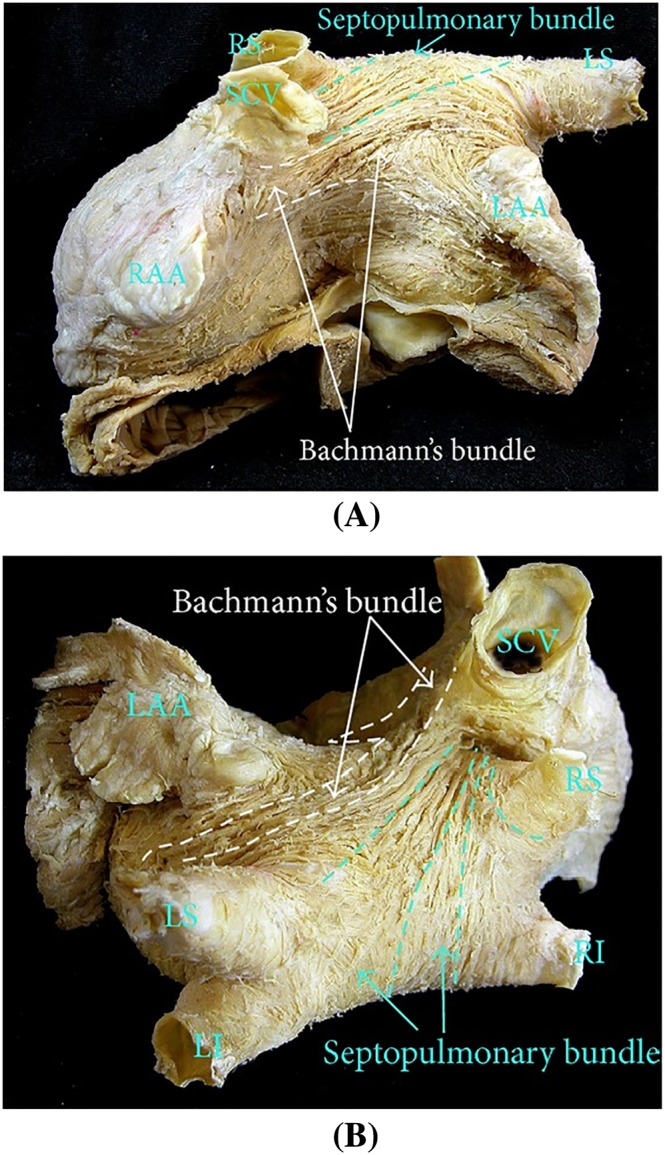
The left atrium fibre structure in the normal human heart. (A) Anterior view, where Bachmann's bundle runs from atrial septum to left atrial appendage, and the septopulmonary bundle runs obliquely to the superior wall. (B) Superior view where the septopulmonary bundle fans out. SCV, superior cava vein; RAA, right atrial appendage; LAA, left atrial appendage; LI, left inferior pulmonary vein; LS, left superior pulmonary vein; RI, right inferior pulmonary vein; RS, right superior pulmonary vein. The figure is from the work by Sánchez‐Quintana et al^32^

**Figure A2:**
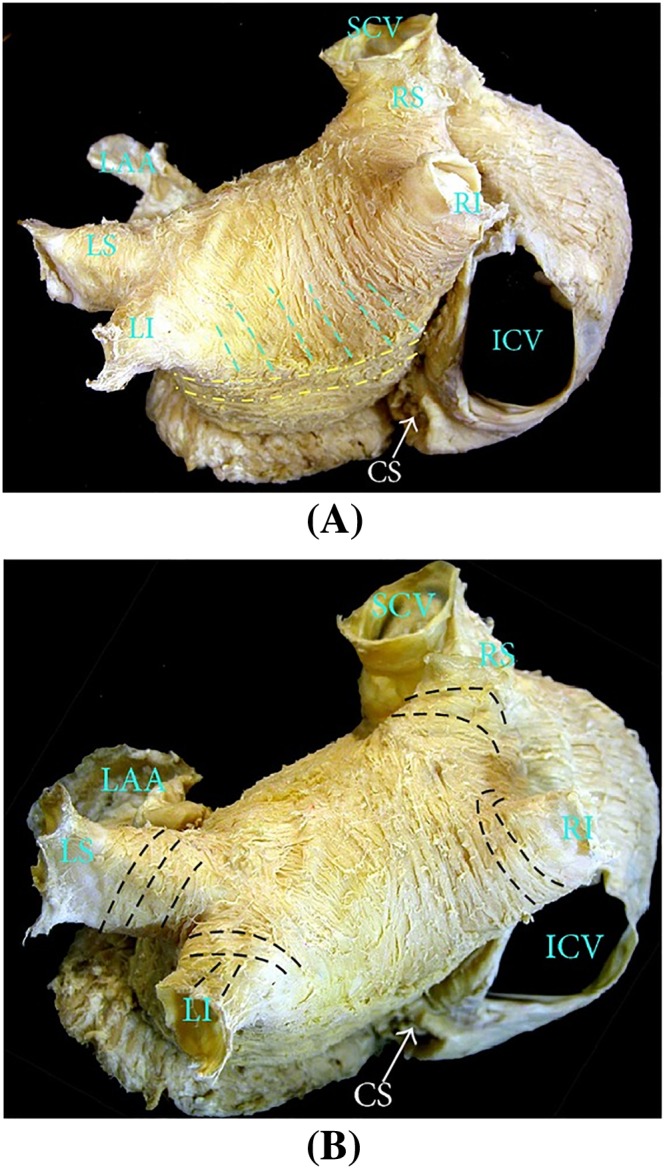
The left atrium fibre structure in the normal human heart. (A) Posterior view, where the septopulmonary bundle meets the circumferential fibres coming from the lateral wall. (B) Near the pulmonary veins insertions where circular fibres are dominant and longitudinal, oblique fibres are also common. ICV, inferior cava vein; CS, coronary sinus. The figure is from the work by Sánchez‐Quintana et al^32^

**Figure B3:**
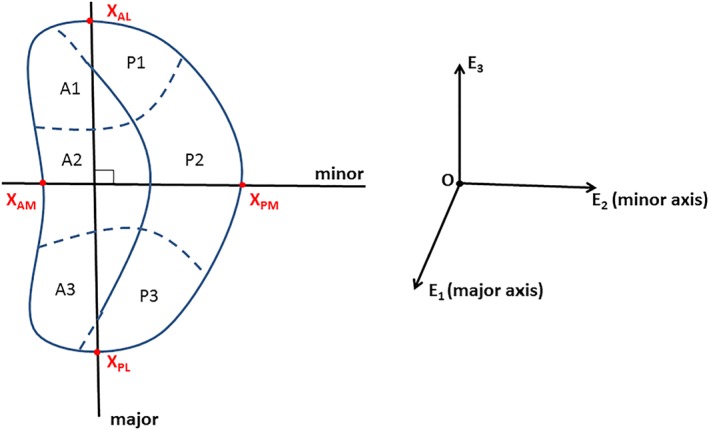
The major and minor axis for mitral annulus demonstrated on a typical D‐shaped annular geometry. A1‐A3 and P1‐P3 are the MV leaflets segments. ***X***_AL_ and ***X***_PL_ are preselected to define the major axis. ***X***_AM_ is the anterior middle point and ***X***_PM_ is the intersection point of ***E***_2_‐***E***_3_ plane and the posterior annulus. ***E***_3_ is the third axis orthogonal to the major‐minor plane. A1, anterolateral segment; A2, anteromiddle segment; A3, anteromedial segment; P1, posterolateral segment; P2, posteromiddle segment; P3, posteromedial segment

## APPENDIX A ATRIAL FIBRE STRUCTURE

Both Ho et al^1^ and Sánchez‐Quintana et al^32^ have shown a detailed description of LA fibre structure, shown in Figures A1 and A2. From the epicardial aspect, the main fibre pattern on anterior wall is represented by Bachmann's bundle, also known as the interatrial bundle, which originates from the junction of right atrium and the superior caval vein, joined by a broad band of circumferential fibres, and runs passing either side of the neck of LAA to the left lateral wall and inferior part of the posterior wall. The septopulmonary bundle represents the group of fibres that comes from anterosuperior septal raphe (septal area marked epicardially by the interatriai groove), underneath the circumferential fibres, runs obliquely to the superior wall and fan out. Near the pulmonary veins, the septopulmonary bundle blends with the circular and longitudinal fibres at the insertions. On the posterior wall, two branches of the septopulmonary bundle are seen: one goes leftward and join the circumferential fibres on the lateral wall; one goes rightward to the posterior septal raphe. From the endocardial aspect, the dominant fibre pattern is described by the septoatrial bundle^81^ which originates from the anterior interatrial raphe and ascend to the superior wall together with the longitudinal fibres from vestibule and blend with the septopulmonary bundle. It also goes leftward and join the circumferential fibres on lateral and posterior wall.

The rule‐based fibre defining method in the current study is summarized as follows: First, the surface fibres are defined within the six sections at LA epicardial surface shown in Figure 2C,D. For each section, the boundary follows the three‐dimensional spline generated by manually selected boundary points and used to divide the LA surface in SOLIDWORKS (Dassault systemes, USA). The pulmonary veins sections are defined to have radial width of approximately 0.5 cm at the insertions to incorporate circular fibre structure. The LAA section is separated simply at the LAA root. The MV plane section is defined with longitudinal width of 1 cm at septal, anterior, and posterior walls, and approximately 2 cm at the lateral wall. The septopulmonary section is defined with oblique boundary lines at anterior and posterior walls to match the fibre morphology observed in Figures A1 and A2. The Bachmann's bundle section is defined to be the rest of the anterior wall.

For each section, a cylindrical coordinate system is defined with longitudinal, radial, and circumferential directions. The surface fibres are defined to follow the circumferential direction and out‐of‐plane fibres are projected onto the tangential plane of the epicardial surface. Finally, the fibre orientations for the volumetric mesh elements are defined using a nearest neighbour approach.

## APPENDIX B MODEL CONNECTION

Since the LA and MV geometries used here are from different patients. It is therefore necessary and important to properly connect two models. The presented approach is based on the mitral annular geometry from both LA and MV models.

As illustrated in Figure B3, for both LA and MV models, we first define the major axis by selecting two annular points at anterolateral and posterolateral side, denoted by ***X***_AL_ and ***X***_PL_. Then the anterior middle point, ***X***_AM_, is chosen at anterior annulus and used to define a plane orthogonal to the major axis. The posterior middle point, ***X***_PM_, is obtained via the intersection of this plane with the posterior annulus. The minor axis is therefore defined by ***X***_AM_ and ***X***_PM_.

The connection of the LA and the MV starts with overlapping the MV annulus central points ***O***_LA_ and ***O***_MV_. Then the MV is rotated to ensure the alignment of major and minor axis with the LA. Next, the MV is shifted along the ***E***_3_ axis to avoid the intersection of two geometries. The gap between the LA and the MV is filled with a rigid housing structure. In the current model, the LV is represented by a rigid tube with ventricular pressure applied at one end and the other end connecting the LA at the MV exclusion. During the ventricular systole, the rigid housing is used to anchor the MV model and bear the high LV pressure.
